# Pregnancy complications and childhood mental health: is the association modified by sex or adverse social circumstances? Findings from the ‘growing up in Ireland’ national infant cohort study

**DOI:** 10.1007/s00127-024-02678-2

**Published:** 2024-04-29

**Authors:** Emma Butler, Mary Clarke, Michelle Spirtos, Linda M O’ Keeffe, Niamh Dooley

**Affiliations:** 1 Dept of Psychology, School of Population Health, Royal College of Surgeons Ireland, Dublin, Ireland; 2 Dept of Psychology, School of Population Health & Dept of Psychiatry, Royal College of Surgeons Ireland, Dublin, Ireland; 3 Dept of Occupational Therapy, Trinity College Dublin, Dublin, Ireland; 4 School of Public Health, University College Cork, Cork, Ireland & MRC Integrative Epidemiology Unit & Population Health Sciences, Bristol Medical School, University of Bristol, Bristol, UK; 5 Centre for Rheumatic Diseases, School of Immunology & Microbial Sciences, Kings College London, UK & Dept of Psychiatry, Royal College of Surgeons Ireland, Dublin, Ireland

**Keywords:** Pregnancy complications, Child mental health, Adverse social circumstances, Sex differences, Prevention

## Abstract

Specific pregnancy complications, socioeconomic position and sex have all been independently associated with child mental health outcomes, but their combined effects remain unclear. We examined whether total number of complications experienced in the pregnancy associated with mental health at 5 and 9-years, and whether this varied by sex or adverse social circumstances. Pregnancy complications were self-reported at 9-months post-natally from a list of 16 complications. Parents completed the Strengths and Difficulties Questionnaire (SDQ) when their child was 5 and 9-years. The primary outcome was the SDQ-total and scoring in the clinical range (> 16) was a secondary outcome. We applied generalized linear mixed models to a large nationally representative Irish cohort (GUI; n = 11,134). Analyses were adjusted for sex, adverse social circumstances (at 9-months), and gestational smoking. We included an interaction term between pregnancy complications and each variable respectively in separate models to examine if associations varied by sex or adverse circumstances.

After controlling for covariates, total complications associated with mental health at 5 and 9-years. Each additional pregnancy complication conferred a 10% higher total-SDQ score (exponentiated co-efficient 1.10 [95%CI 1.06–1.14], 1.20 [1.15–1.26], 1.20 [1.12–1.29] and 1.34 [1.21–1.48] for 1, 2, 3 and 4 + complications respectively). For the dichotomised outcome, generally increasing odds for clinical levels of mental health difficulties were observed (OR 1complication = 1.89, 95%CI [1.37–2.59]; OR 2complications = 2.31, 95%CI [1.53–3.50]; OR 3complications = 1.77, 95%CI [0.89–3.52]; OR 4 + complications = 6.88, 95%CI [3.29–14.40]). Females had significantly lower odds of exhibiting clinically significant mental health difficulties than males (OR = 0.43, 95%CI[0.32–0.57]).

There was no evidence that the association between pregnancy complications and child’s mental health varied by sex or social circumstances at 5 or 9-years. Males exposed to numerous pregnancy complications in the context of adverse social circumstances had the highest predicted probability of having mental health difficulties in middle childhood.

**Supplementary Information:**

The online version contains supplementary material available at 10.1007/s00127-024-02678-2.

## Introduction

The developmental origins of mental illness are incompletely understood [1]. Given that a substantial amount of brain development takes place in-utero, the prenatal period is a time of particular susceptibility to environmental influences [2]. Numerous specific pregnancy complications such as hypertension, bacterial infection, anaemia, influenza and gestational diabetes mellitus have been associated with different diagnoses in offspring such as psychosis, schizophrenia, bipolar disorder and attention deficit hyperactivity disorder (ADHD) [3–7] but with small effects. Yet these individual pregnancy complications rarely exist in isolation [8, 9] and as a result, the strength of any associations identified may change if the total number of pregnancy complications (PCs) that occur in-utero is considered.

Studies that have considered total number of complications experienced in a pregnancy in relation to that child’s mental health have revealed mixed findings and tend to include birth complications in their cumulative measure i.e. include exposures that occurred both in-utero and during the birthing process [10–16]. For example, Wagner et al.(2009)found that a higher number of obstetric/neonatal complications did not increase the odds of ADHD or conduct disorder [15] at 7–8 years (n = 750 twins) but Milberger et al.(1997) found that the total number of complications was positively associated with an ADHD diagnosis among males aged 6–17 years (n = 260) [16]. Fuchs et al. (2022) found that the total cumulative obstetrical complications predicted Total Problems Score on the Child Behaviour Checklist (CBCL) at 8-years in a very small sample (n = 54) [10]. In a much larger sample (n ~ 7898), the number of adverse prenatal exposures was associated with the odds of clinically elevated CBCL scores at 9–10 years [11]. The effect was only significant when two or more adverse prenatal exposures occurred, and a dose-response association existed between the number of prenatal exposures and the odds of clinically elevated CBCL scores. One potential explanation for the mixed findings is the different combination of pre- and perinatal risks that these studies consider. Some of the cumulative scores included birth complications (e.g. gestational age, mode of delivery, low birth weight) however birth complications could be effect modifiers or mediators of the association between complications in pregnancy and child outcomes. In fact, it has been demonstrated that pregnancy complications significantly differed by gestational age [17]. Therefore, the focus of this study is on complications that occur in-utero *prior* to birth.

As to whether the effects of exposure to pregnancy complications on mental health outcomes varies by sex or the socio-economic status of the child is also unclear. Laucht et al. (2000) found no evidence that the association between number of pregnancy complications and child mental health (measured by total-CBCL score at 8-years) differed by sex or adverse family circumstances [18]. Wagner et al. (2009) reported a stronger association between cumulative perinatal score and ADHD/conduct disorder for females compared to males at 7/8-years [15], and Nomura et al. (2012) reported that the effect of gestational diabetes on ADHD did differ by socioeconomic status at 4–6 years [7]. Inflammation markers in pregnancy were found to be associated with internalising problems in females but not males at 9–11 years [19] whilst bacterial infection in pregnancy (n = 15,421) was more strongly associated with psychosis in males over 40-year period [4]. Studies examining these interaction effects are however extremely limited in the literature on cumulative pregnancy complications and outcomes in middle childhood (5–12 years).

Furthermore, examining the effects of pregnancy complications on one specific mental health diagnosis at one point in time has limited practical utility in paediatric populations, as children tend to move within, and across, different diagnostic mental health categories [20–23]. A more general measure of observed emotional and behavioural problems may therefore be a better way to capture poor mental health in young childhood compared to specific binary disorders [24, 25]. The p-factor representing the person’s general psychopathology vulnerability is an important indicator of current childhood psychopathology and a good predictor of later mental health [23].

The current study explored the association between cumulative pregnancy complications and childhood mental health at 5 and 9-years in a large population-based sample of Irish children. We aimed to investigate: (1) whether a dose-response relationship existed between the total number of pregnancy complications experienced and later mental health problems; (2) whether cumulative pregnancy complications were associated with *clinically* significant mental health scores; and (3) whether any of these associations differed by sex or adverse social circumstances.

## Methods

### Data source

This is a secondary analysis of data from the on-going Growing Up in Ireland (GUI) infant study, a prospective cohort of children recruited nationally in 2008 at 9-months old (n = 11,134). The data is openly accessible to those affiliated with Irish institutions. We used data from three waves (report of pregnancy complications & social circumstances from wave 1, and SDQ-scores from waves 3 and 5).

Children were sampled from the Child Benefit Register and stratified by marital status, county of residence, nationality and number of children in household to achieve national representativeness [26]. The proportions of unemployed parents, non-nationals, specific maternal age groups, and parental education levels in the baseline GUI sample approximated those in the Irish population, thus the cohort is generally representative of the range of demographic and socioeconomic diversity of Ireland [27]. A weighting variable is available to counteract attrition over time. Further details on the sample design, recruitment procedures, ethics, methodology and findings here (https://www.growingup.gov.ie/). This secondary analysis was approved by the Research Ethics committee for the Royal College of Surgeons Ireland. (RCSI RIMS 212,610,659)

## Measures

### Exposure

#### Pregnancy complications (PCs)

At 9-months post-natal, mothers retrospectively reported if they had experienced any of the following 16 pregnancy complications: placenta problems, pre-eclampsia, urinary tract infection (UTI), persistent vomiting, late bleed, vaginal infection, intrauterine growth restriction (IUGR), Rhesus negative, influenza, ‘other’ blood problems, ‘other’ pains, ‘other’ high/moderate risk condition, maternal condition not thought to affect the fetus, gestational diabetes mellitus (GDM), blood pressure problem but not pre-eclampsia, ‘other’. Total number of complications was calculated for each mother and subsequently categorised 0, 1, 2, 3 or 4+.

### Outcome

#### Strengths and difficulties questionnaire (SDQ)

Childhood mental health was measured using the SDQ [28], completed by the primary caregiver when the child was 5 and 9-years old. The SDQ is a valid and reliable instrument to screen for emotional and behavioural problems in children aged 3–16 years and is widely used in research and clinical practice [29]. The SDQ is a parent-rated questionnaire containing 25-items on a 3-point likert scale (0 = not true; 1 = somewhat true; 2 = certainly true), five items are reverse scored. Item scores are aggregated into 5 subscales. The first four subscales combine to calculate a total difficulties score ranging from 0 to 40. Higher scores indicate higher difficulties. SDQ-total at 5-years and/or 9-years was the primary outcome. For the secondary outcome, the children were categorised into two groups based on their total-score at the recommended cut-offs (sdqinfo.org). Scores of 17 or above were considered to be in the clinical range. Goodman et al. (2001) showed that parent-reported SDQ-total scale has higher internal consistency (Cronbach’s-alpha = 0.82) and test-retest reliability than the four subscales [30].

### Covariates

#### Adverse social circumstances

The cumulative risk hypothesis asserts that the accumulation of risk factors, independent of the presence or absence of particular risk factors, impacts child outcomes, such that the greater number of risk factors, the greater the prevalence of clinical problems [31–36]. Therefore, we created a social risk (SR) score based on post-natal sociodemographic characteristics of the child’s environment at 9-months old guided by the method of Li et al. (2020) comprising of maternal age, education, relationship status, race and family income quintile [37]. Both higher (36 + years) and lower maternal age at birth (15–25 years) were considered risks. Due to the lack of variability in race in the dataset, migrancy of the mother was used, with mothers’ birth outside of Ireland considered a risk. Family income reflected that from all sources, after tax and social insurance, divided by the number of people in the household. A 3-category variable was created from quintiles to denote high (Q4&5), middle (Q3) and low income (Q1&2). Maternal education had 4 levels: (1) no formal education, up to and including any level of secondary school education, (2) post-secondary training up to and including diploma, (3) degree-level and (4) post-graduate (MSc/PhD) with less education corresponding to higher risk scores. Lastly, being married/living with a partner was considered low risk, and single/widowed/divorced/separated/never married considered higher risk. Values were then added and divided into four social risk categories: none, low, moderate and high (Table S.1a). Further details of how SR groups were created are in the supplementary material. These SR categories aligned well with primary caregiver response to the question “how easy/difficult is it for the household to make ends meet?” concurrently at 9-months and prospectively at 5 and 9-years (Table S.1b).

#### Smoking in pregnancy

Exposure to smoke in pregnancy was assessed by the maternal response to “including yourself, how many people in the household smoked whilst you were pregnant?” using the questionnaire administered at 9-months.

### Participants included in analyses

GUI had participation rates of 80.8% at 5-years and 72.1% at 9-years. Participants were included if (1) mother was the respondent at baseline (n = 38 excluded) (2) pregnancy data was available (n = 1 excluded) (3) they had an SDQ-total at *either* 5 or 9 years (n = 1794 excluded) (4) covariate data (adverse social circumstances and smoking in pregnancy was available (n = 818 excluded). Consequently, data from 8,483 children was analysed (n = 7025/8483 had both 5 and 9-years SDQ available). Fig. S1 provides participant flow-chart.

Attrition analysis were conducted to test whether participants whose data were analysed (n = 8483) were different on important background characteristics from non-participants at follow-up (n = 2651). Briefly, analysed children’s mothers: reported slightly more pregnancy complications, were more likely to be born in Ireland, had slightly higher levels of education and family income and thus had slightly lower levels of cumulative social risk. All descriptive and inferential results were therefore weighted to account for sampling bias. There was no substantially meaningful differences on any other variables of interest (Table S.5).

## Data analysis

Analyses were performed in STATA (Stata, v.17). For descriptive statistics, a table of the distribution of the covariates across the pregnancy complication categories was created. All analyses were also stratified firstly by sex and subsequently by SR.

To examine the association between pregnancy complications and SDQ-total, we used Generalised linear mixed models (GLMMs) with a random effect of participant ID. This allowed us to account for multiple observations for some participants and an alternative distribution and link function accounted for the positive skew in SDQ-total (i.e. not normally distributed as most children scored low). This meant that participants could be included if they provided an SDQ-score at one time (age 5/9) or both.

We demonstrated, using goodness-of-fit statistics that a mixed-model with gamma-distribution and log-link function was the best fit to the data (Table S.2a). Stages in model selection were: (1) intercept-only; (2) fixed-effects of pregnancy complications only; (3) random-effects only; (4) fixed and random effects; (5) sex; (6) SR; (7) full model including smoking in pregnancy. The full model demonstrated the best model fit.

Effects are modelled on ‘change in log mean’ but results are transformed and provided as exponentiated coefficients for easier interpretability. Post-hoc comparisons between all possible levels of exposures of interest were conducted to test all possible contrasts. The ‘Scheffe’ adjustment for multiple comparisons was used as it is the most conservative adjustment with widest confidence intervals (CIs) but at a cost of the lowest power. For a gamma distribution all response values must be > 0. Therefore the outcome was winsorized with n = 532 (6.3%) participants being assigned an SDQ-total of 1 rather than 0 (max. possible total = 40). Relevant descriptive statistics were calculated to ensure that this change did not significantly alter the mean, median, standard deviation or inter-quartile range (Table S.2b).

To examine whether pregnancy complications were associated with clinically significant SDQ-totals, a GLMM specifying binomial distribution and its default link function was used. The stages involved and conclusions in model selection were the same as in aim 1(Table S.3a). Results are reported as Odds Ratios. The predicted probabilities for being in the clinical range for different combinations of predictors were also calculated (Table S.4).

To examine whether the effect of pregnancy complications on childhood mental health differed by sex or adverse social circumstance (SR) we added a two-way interaction term (complications*sex; complications*SR) to separate fully-adjusted GLMMs.

## Results

### Sample characteristics

As can be seen from Table 1, the most commonly reported pregnancy complication was persistent vomiting and urinary tract infections followed by blood pressure problems and pre-eclampsia. Whilst many of the complications had low prevalence, the *total number of complications* was the primary exposure of interest in this study.

**Table 1:** Prevalence of specific pregnancy complications reported, total number of pregnancy complication groups and SDQ outcome for the whole sample, followed by split by sex, followed by split by social risk group

	Total	Split by sex		Split by social risk group			
		Male	Female	None	Low	Mod	High
N=	8483	4277	4206	1551	2901	2568	1463
Variable	(% yes)	(% yes)	(% yes)	(% yes)	(% yes)	(% yes)	(% yes)
Placenta complication	3.24	3.2	3.28	3.82	2.81	2.64	4.2
Pre-eclampsia	7.5	7.45	7.53	5.06	7.57	7.18	9.23
^a^UTI	15.17	15.1	15.25	10.2	13.74	15.1	20.0
Hyperemesis	17.64	15.96	19.4	14.38	17.35	17.35	20.34
Late bleed	6.7	7.42	5.94	4.76	5.46	5.94	10.35
Vaginal infection	3.44	3.14	3.75	3.45	2.88	3.65	3.85
^b^IUGR	2.24	2.33	2.14	1.24	2.31	1.45	3.75
Rhesus	3.67	3.49	3.86	5.24	3.95	3.3	2.87
Influenza	3.76	3.27	4.27	3.45	3.86	3.23	4.51
‘Other’ blood	2.29	2.02	2.56	1.39	2.14	2.6	2.6
‘Other’ pains	2.44	2.29	2.6	2.7	2.88	2.47	1.71
‘Other’ high/mod risk condition	2.39	2.15	2.65	3.24	2.92	1.92	1.86
Maternal condition not thought to affect fetus	2.83	2.87	2.79	3.45	3.22	2.72	2.12
^c^GDM	3.0	2.82	3.2	1.7	2.17	4.12	3.36
^d^BP problem but not PE	10.32	10.0	10.71	9.46	10.74	9.44	11.47
‘Other’	1.2	1.07	1.33	1.29	1.17	1.31	1.02
Total no. of pregnancy complications:							
0	44.91	46.81	42.93	48.36	45.67	46.1	40.37
1	33.61	32.67	34.59	35.32	33.41	32.62	34.12
2	14.02	13.1	15.0	11.35	13.95	14.57	14.99
3	4.76	5.06	4.45	3.4	4.62	4.7	5.82
4+	2.7	2.38	3.03	1.57	2.36	2.01	4.7
SDQ-total @5	7 (4-10)	7 (4-11)	6 (4-10)	6 (3-9)	6 (3-9)	7 (4-10)	8 (5-13)
Median(IQR)							
SDQ-total @9	6 (3-10)	7 (4-12)	6 (3-10)	5 (3-9)	6 (3-10)	7 (4-11)	8 (4-13)
Median(IQR)							

^a^Urinary tract infection. ^b^Intrauterine Growth Restriction. ^c^Gestational Diabetes Mellitus. ^d^Blood pressure problem but not pre-eclampsia

Almost 45% of mothers experienced no pregnancy complication, 33.6% had one complication, 14% had two and 7.5% experienced 3+ (Table 1). At 5-years, 5.9% of the sample had SDQ-totals considered to be in the clinical range increasing to 8.3% by 9-years (Table 2). Total number of complications was associated with markers of social risk (income, maternal education and single parenthood) and increasing proportions of children in the clinical range and median SDQ-totals (Table 2).

**Table 2:** Descriptive characteristics of the sample according to total number of pregnancy complications reported. (All variables apart from the outcome were reported at 9-months)

	Total (n=8483)	No PC	One PC	Two PCs	Three PCs	4+ PCs
		(n=3945)	(n=2817)	(n=1136)	(n=375)	(n=210)
Sex (% male)	51.05	53.21	49.63	47.62	54.22	45.06
SDQ-total @5-years (med, IQR)	7(4-10)	6(4-10)	7(4-11)	7(4-11)	7(4-11)	10(5-16)
SDQ-total @9-years (med, IQR)	6(3-10)	6(3-10)	7(4-11)	7(4-12)	8(4-12)	8(5-14)
SDQ@5-y (%clinical)	5.9	4.21	6.49	6.73	7.19	20.23
SDQ@9-y (%clinical)	8.31	6.61	8.7	9.6	11.69	19.14
Maternal age in years (Med, IQR)	32(28-35)	32(28-36)	32(28-36)	31(27-35)	32(27-35)	29(25-35)
Migrancy (%yes)	18.95	19.8	18.66	16.29	22.87	15.36
^a^Family income quintile (%):						
High	38.6	39.86	39.44	35.38	35.66	29.24
Med	20.08	19.94	19.2	24.09	18.43	15.66
Low	41.31	40.21	41.36	40.53	45.9	55.11
Maternal education level (%):						
MSc/PhD	10.97	11.8	11.68	8.55	8.84	4.59
Degree	16.44	18.48	15.77	13.95	11.2	12.95
Post-secondary up to diploma	27.87	26.41	29.11	27.82	32.17	29.3
None up to secondary	44.73	43.3	43.45	49.68	47.8	53.16
#smoke in house in pregnancy:						
0	64.94	65.54	66.17	61.95	64.1	56.51
1	25.09	24.73	24.33	27.01	26.6	28.04
2	8.33	7.93	8.08	9.8	6.96	12.89
3	1.64	1.8	1.43	1.24	2.34	2.56
Mother in relationship (%no):	16.1	13.43	17.19	18.62	16.65	33.07
^b^Social risk category (%):						
None	14.55	15.67	15.29	11.79	10.4	8.44
Low	29.76	30.26	29.58	29.61	28.89	25.96
Moderate	31.44	32.27	30.51	32.67	31.06	23.38
High	24.25	21.8	24.62	25.93	29.65	42.22

^a^Family Income Quintiles reflected that from all sources, after tax and social insurance divided by the number of people in the household. These quintiles were then collapsed into three categories with quintile 1&2 considered ‘low’, quintile 3 considered ‘middle’ and 4&5 considered ‘high’ income

^b^See methods for construction, represents maternal age, education level, migrancy, family income and mothers relationship status when baby was 9-months old

Table 3 shows a small but statistically significant relationship between cumulative pregnancy complications experienced and SDQ-total, which remained significant after inclusion of covariates in a dose-response fashion. The exponentiated co-efficient is the % change increase in SDQ-total compared to those with no complications. Fully adjusted models suggested children who experienced 4 + complications have a 34% increase in total-SDQ compared to children who experienced no complications. In real terms, this means that children with 4 + complications have SDQ-total scores that are 2-points higher than children with no pregnancy complication. Post-hoc adjusted comparisons showed a significant difference between any level of complications with none, and between 2 pregnancy complications and 1 complication.

**Table 3:** Association between total number of pregnancy complications on SDQ-total at 5 and 9-years, adjusted for all covariates and stratified by sex and adverse social circumstances (SR). Shown are exponentiated beta coefficients (& 95%CIs)

	Effect estimates and 95% CI’s after adjustment						
	Total	Stratified by sex		Stratified by ^a^social risk			
Total no.of ^b^PCs		Male	Female	No risk	Low risk	Mod risk	High risk
	(n=8483)	(n=4277)	(n=4206)	(n=1551)	(n=2901)	(n=2568)	(n=1463)
None	Reference						
1	1.1	1.12	1.07	1.12	1.05	1.1	1.17
	(1.06-1.14)	(1.07-1.18)	(1.02-1.13)	(1.04-1.21)	(0.99-1.12)	(1.03-1.17)	(1.06-1.28)
2	1.2	1.25	1.16	1.28	1.2	1.16	1.17
	(1.15-1.26)	(1.17-1.33)	(1.08-1.23)	(1.15-1.43)	(1.11-1.30)	(1.06-1.26)	(1.05-1.31)
3	1.2	1.24	1.16	1.27	1.15	1.19	1.23
	(1.12-1.29)	(1.13-1.37)	(1.05-1.28)	(1.07-1.50)	(1.02-1.30)	(1.05-1.36)	(1.05-1.44)
4+	1.34	1.46	1.24	1.22	1.31	1.27	1.47
	(1.21-1.48)	(1.26-1.69)	(1.08-1.42)	(0.93-1.60)	(1.10-1.56)	(1.05-1.55)	(1.22-1.78)

^a^ See methods for construction, represents maternal age, education level, migrancy, family income and mothers relationship status when baby was 9-months old

^b^Pregnancy complications

Table 4 shows a statistically significant general increase in the odds of having SDQ-total scores in the clinical range relative to number of complications experienced. In the fully-adjusted model, children with 2 complications had two-fold increased odds of clinically significant SDQ-total scores (OR = 2.31), children with 4 + complications had six-fold increased odds of clinically significant SDQ-total scores (OR = 6.88). The association between 3 pregnancy complications on the odds of clinically significant difficulties was non-significant. Post-hoc adjusted comparisons show a significant difference between levels 4+, 2 and 1 complication compared with none and between 4 + and 3 complications.

**Table 4:** Association between total number of pregnancy complications and clinical SDQ category at 5 and 9-years, adjusted for all covariates and stratified by sex and adverse social circumstances (SR). Shown are Odds Ratios (& 95%CIs)

	Effect estimates and 95% CI’s after adjustment						
	Total	Stratified by sex		Stratified by ^a^social risk			
Total no. of ^b^PCs		Male	Female	No risk	Low risk	Mod risk	High risk
	(n=8483)	(n=4277)	(n=4206)	(n=1551)	(n=2901)	(n=2568)	(n=1463)
None	Reference						
1	1.89	2.65	1.18	1.84	1.45	2.16	2.24
	(1.37-2.59)	(1.72-4.10)	(0.74-1.89)	(0.97-3.51)	(0.87-2.39)	(1.20-3.89)	(1.01-4.93)
2	2.31	3.23	1.5	4.06	1.82	2.89	1.2
	(1.53-3.50)	(1.83-5.69)	(0.82-2.75)	(1.92-8.56)	(0.98-3.40)	(1.34-6.21)	(0.43-3.38)
3	1.77	2.27	1.25	1.24	0.92	2.59	2.44
	(0.89-3.52)	(0.90-5.69)	(0.44-3.58)	(0.16-9.73)	(0.32-2.61)	(0.74-9.12)	(0.49-12.04)
4+	6.88	10.73	4.23	3.84	3.29	10.6	9.8
	(3.29-14.40)	(3.73-30.86)	(1.51-11.83)	(0.98-15.08)	(1.12-9.66)	(2.67-42.11)	(1.80-53.42)

^a^ See methods for construction, represents maternal age, education level, migrancy, family income and mothers relationship status when baby was 9-months old

^b^ Pregnancy complications

**Fig. 1:**
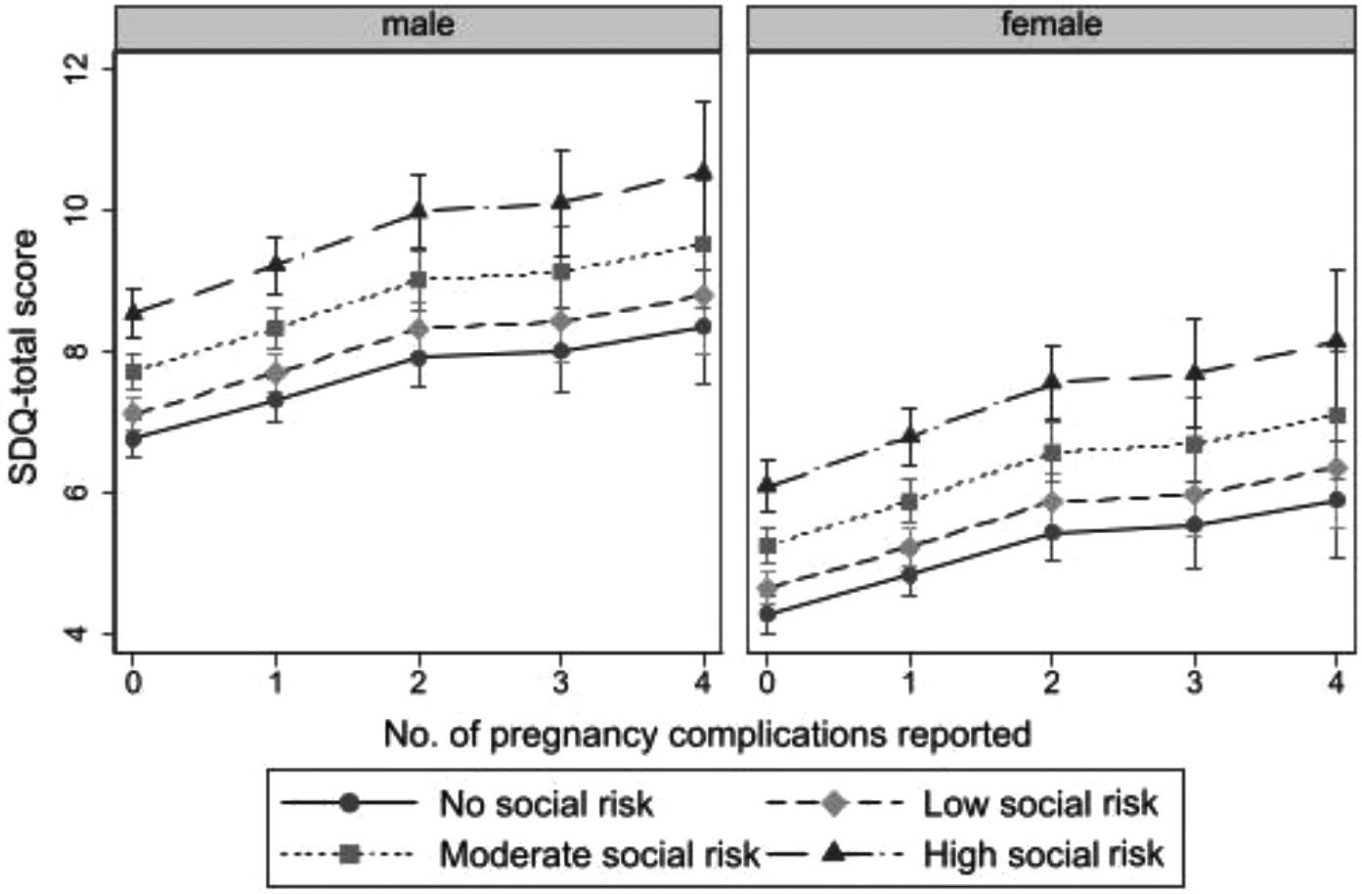
Predicted SDQ-total (with confidence interval) for males and females according to number of pregnancy complications experienced and level of cumulative social risk (maternal age, education, relationship, migrancy and family income) adjusted for gestational smoking

In order to test whether there was a multiplicative interaction between pregnancy complications and sex, and pregnancy complications and SR, an interaction term was added to each fully adjusted model. Model fit was assessed using AIC, BIC, LR & Chi^2^. Neither interaction term was statistically significant nor improved model fit indicating no evidence for multiplicative interaction (Table S.6). Despite this, significant main effects of sex and social risk were observed on SDQ-totals. In fully-adjusted models, females had 16% significantly lower SDQ-total than males and social risk (SR) had a dose-response relationship with SDQ-total (Fig. 1). Post-hoc adjusted comparisons showed significant difference between all levels of SR pairwise comparisons with the exception of low SR and none.

Similarly, the odds of having *clinically* significant difficulties were 57% lower (95% CI 43–68%) in females compared to males. Social risk exhibited its own dose-response relationship with odds of having clinically significant difficulties (Table S.7). Post-hoc adjusted comparisons showed significant differences between all levels of SR pairwise comparisons except for low SR with none.

Additionally, despite there being no evidence of a moderation effect between pregnancy complications and social risk, their combined effects appeared to be additive. This was evident from the average predicted probability of having clinically significant mental health difficulties for a male with 4 + complications and high SR being 22.6% (95%CI 15.4–29.7%) compared to a female with no complications & no SR being 2.1% (95%CI 1.6–2.6%) (Table S.4).

## Discussion

This study is one of few prospective studies using national cohort data to investigate *cumulative* pregnancy complications and child mental health outcomes. We found that children of mothers who experienced more complications had poorer mental health in middle childhood than children of mothers with no complications in a dose-response fashion. The majority of women (78.7%) experienced none or only one pregnancy complication. However, 21.3% of women experienced 2 or more complications, and their children had double to six-fold increased odds of experiencing clinically significant levels of mental health symptoms in middle childhood compared to children whose mothers experienced no complications. This is similar to the finding of Dooley et al. (2023) who found that cumulative number of complications was a predictor of attention problems in a U.S. cohort [38] and a dose-response relationship was also identified between cumulative number of prenatal exposures and child psychopathology in the U.S. cohort [11].

There are at least two mechanisms discussed in the literature that could explain the relationship between cumulative complications and child mental health, namely “allostatic load” [39] and immune/inflammatory processes on the developing brain [40, 41]. Although we cannot infer a causal relationship and it may not be possible to disentangle the myriad of causal possibilities underlying the association between pregnancy complications and child mental health, it could be considered as a possible prognostic factor for child mental health. Our findings highlight the potential of the cumulative number of complications experienced to be used to screen/identify children at-risk of poorer mental health outcomes who may benefit from early intervention.

This is of public health concern for two reasons. From an economic perspective, the costs of intervening for families at-risk preventatively has been shown to be lower and require less intense treatment that the cost of providing interventions targeting children who already present in the clinical range [42]. Screening for pregnancy complications and SR at baby developmental appointments may help identify vulnerable individuals during a unique window of opportunity for early interventions, promoting both maternal and child well-being *prior* to the onset of mental health difficulties. Secondly, although we know there is a relationship between social-risk and outcomes such as cognition in high-risk groups e.g. preterm birth [43], our findings highlight a relationship between social-risk and *mental health* outcomes at population-level, that is, for *all* children.

In fact, both pregnancy complications and social risk exhibited independent but similar magnitudes of association with child mental health. O’ Callaghan et al. (1997) similarly found that maternal age, education, family income and marital relationship at the time of first visit in pregnancy were significantly associated with child behaviour scales at 5-years [44]. Unlike our findings, their common obstetric and perinatal risk factors did not independently predict child behaviour problems in children at 5-years once adjusted for social disadvantage. However they did not consider them *cumulatively.* Our finding that both cumulative complications and SR were both independent and significant risk factors point towards the possibility that low-cost, non-invasive, easily measurable factors in the perinatal period may be useful in terms of predicting and identifying children at-risk of poor mental health outcomes *before* symptoms are evident.

Whilst we did not find evidence to support significant quantitative interaction for child sex, similar to other studies [20], males exhibited a stronger dose-response effect compared to females. Sex-based differences in brain development and responses to insults in-utero are still not fully understood. Some studies have shown associations between placenta size and mental health outcomes in males only [45] whilst others have shown associations between prenatal predictors and childhood depression/anxiety in females only [46]. A viability-vulnerability trade-off has been suggested with males possibly being more vulnerable to in-utero in the shorter term but with females possibly adapting and feeling the consequences of this adaptation in the longer term [47]. This may explain why in our study males were found to be more at-risk as our outcome was measured at 5 and 9-years of age and females have been shown to experience mental health symptoms later in development compared to males [48–52].

It is important to note that although there was increased risk among children exposed to complications, the majority of children (ranging from 80 to 95%) of mothers with complications did not have mental health problems. Further research into determinants of such resilience might be important for designing interventions for children at-risk. Additionally, future research should consider cumulative exposures in pregnancy across biopsychosocial domains not just biological, psychological or social exposures independently. Furthermore, in line with more recent research [53], the strength of pre-conception experiences as prognostic factors should also be considered as this would allow us to shift the window of intervention even earlier in the life-course.

### Strengths & limitations

We used a standardised well-validated instrument (SDQ) as a measure of child’s mental health at two time-points in middle childhood. A 2021 study empirically tested parents potential reporting biases due to their own psychopathology in a large sample and found only minimal evidence for biases in maternal reports of child psychopathology [54]. Repeated measures at two time-points increases reliability also. Mothers’ report of pregnancy complications has been shown to be reliable [55, 56] and was recorded at 9-months post-natally, long before the child’s mental health symptoms were measured.

We measured SDQ-total at two ages but did not specifically look at the change of association (if any) between pregnancy complications and SDQ-total at each time-point. We did not explore whether it is a sub-facet of mental health that is related to pregnancy complications i.e. whether there is a specific effect between pregnancy complications and internalising or externalising problems.

Experiencing 3 complications skewed the overall dose-response relationship (Fig. 1). Whilst this could be attributable to unmeasured confounding it is also possible that sample sizes at higher levels of complications and the need to apply the ‘scheffe’ correction for all possible multiple comparisons may have led to power issues at higher levels of complications. The analysis needs to be replicated with a priori hypotheses to examine whether this was a power issue or a particular pattern of risk/resilience.

Whilst we did conduct a sensitivity analysis (S.8) including maternal depression score at baseline, we are cognisant that we did not have information available pertaining to psychiatric family history. Additionally, information about the use of alcohol/substance use in pregnancy is not available in the data-file and thus has not been included.

This is a longitudinal cohort where some people were lost to follow-up. While we weighted the analysis, there may be residual bias.

## Conclusion

Research into the heritability of neuropsychiatric disorders suggests that while genetic and epigenetic factors play an important role, the manifestation of these disorders is likely to be multi-factorial, involving pre and/or postnatal insults [57]. Our findings indicate that pregnancy complications, social risk and sex all independently contributed to mental health outcomes in childhood.

Our findings support that the total number of complications experienced during pregnancy associated with child mental health outcomes in middle childhood in a dose-response fashion even after adjustment for covariates. Our findings indicate that males who experienced 4 + pregnancy complications in the context of social risk were most at-risk of later psychopathology and therefore may need to be prioritised for infant mental health intervention. The combined presence of early life risks such as pregnancy complications, male sex and high social risk post-natally has important implications for both clinical practice and policy.

## Electronic supplementary material

Below is the link to the electronic supplementary material.

Supplementary Material 1

## Data Availability

No datasets were generated or analysed during the current study.
