# Supplementary material for: Pregnancy complications and childhood mental health: is the association modified by sex or adverse social circumstances? Findings from the ‘growing up in Ireland’ national infant cohort study
Source: Soc Psychiatry Psychiatr Epidemiol. 2024 Apr 29;59(10):1697–707. doi: 10.1007/s00127-024-02678-2 (PMC11464566; doi:10.1007/s00127-024-02678-2)
Supplement: Supplementary file 1 — Supplementary Material 1 [file 127_2024_2678_MOESM1_ESM.docx]

Title: Pregnancy complications and childhood mental health: is the association modified by sex or adverse social circumstances? Findings from the ‘Growing Up In Ireland’ national infant cohort study.

Journal: Social Psychiatry and Psychiatric Epidemiology.

Emma Butler, Dept of Population Health, Royal College of Surgeons Ireland, Dublin, Ireland. [Emmabutler21@rcsi.ie](mailto:Emmabutler21@rcsi.ie) 0000-0002-9769-0591

Mary Clarke, Dept of Psychology & Psychiatry, Royal College of Surgeons Ireland, Dublin, Ireland.0000-0001-5155-9400

Michelle Spirtos, Dept of Occupational Therapy, Trinity College Dublin, Dublin, Ireland. 0000-0002-2246-8910

Linda O Keeffe, Dept of Epidemiology, University College Cork, Cork, Ireland & MRC Integrative Epidemiology Unit, University of Bristol & Population Health Sciences, Bristol Medical School, Bristol, UK

Niamh Dooley, Centre for Rheumatic Diseases, School of Immunology & Microbial Sciences, Kings College London, UK & Dept of Psychiatry, Royal College of Surgeons Ireland, Dublin, Ireland.0000-0003-3161-3996

**Table S.1a**

*The proportion of people in the sample (n=8483, weights applied) who received relevant points towards the cumulative social risk score.*

|  | 0 – No risk (%) | 1 – Low risk (%) | 2 – Mod risk (%) | 3– High risk (%) |
| --- | --- | --- | --- | --- |
| Maternal Age | 59.39 | 40.61 | N/A | N/A |
| Migrancy | 81.05 | 18.95 | N/A | N/A |
| ^a^Family Equivalised Income | 38.60 | 20.08 | 41.31 | N/A |
| Maternal Education Level | 10.97 | 16.44 | 27.87 | 44.73 |
| Maternal relationship status | 83.90 | 16.10 | N/A | N/A |
| Cumulative Social Risk Category | 14.55 | 29.76 | 31.44 | 24.25 |

^a^ Family Equivalised Income reflects that from all sources, after tax and social insurance divided by the number of people in the household.

**Table S.1b**

*Comparison of the derived cumulative social risk score at 9-months with the question “How easy/difficult is it for the household to make ends meet?” at each wave to demonstrate validity (weights applied, n=8483)*

|  | Wave 1 – 9 months  Social Risk: mean(SD) | Wave 3 – 5 years  Social Risk: mean(SD) | Wave 5 – 9 years  Social Risk: mean(SD) |
| --- | --- | --- | --- |
| With great difficulty | 2.48 (.62) | 2.22 (.78) | 2.23 (.79) |
| With difficulty | 2.32 (.71) | 1.91 (.91) | 2.18 (.84) |
| With some difficulty | 1.96 (.88) | 1.69 (.95) | 1.85 (.93) |
| Fairly easily | 1.39 (.98) | 1.33 (1.04) | 1.50 (.98) |
| Easily | 1.20 (1.02) | 1.14 (1.04) | 1.26 (1.02) |
| Very easily | .85 (.93) | 1.02 (1.09) | 1.01 (1.05) |

**Fig. S.1** *Flow-chart of participants*

Excluded:

N=1 no PC information

N=38 respondent was not mother @ baseline

N= 1794 did not have SDQ-total @ 5 OR 9 years.

N= 663 missing cumulative adverse social circumstances

N=155 missing smoking in pregnancy

N= 11, 134 recruited as baseline

N = 8483 included in analysis

(n=7025 have BOTH time-points)

**Table S.2a**

*Demonstrating using goodness of fit statistics that GLMM with gamma distribution and link function was appropriate for the data*

|  | n= | Loglikelihood | df | AIC | BIC | LRchi2(p-val) |
| --- | --- | --- | --- | --- | --- | --- |
| Null Model | 14360 | -43859 | 2 | 87721 | 87736 |  |
| +fixed only (preg) M2 | 14360 | -43773 | 6 | 87558 | 87603 | 171.46 (.000) |
| +random only (id) | 14360 | -42833 | 3 | 85672 | 85695 |  |
| + fixed and random (preg & id) M4 | 14360 | -42775 | 7 | 85564 | 85617 | 115.99 (.000) |
| + sex | 14360 | -42701 | 8 | 85418 | 85479 | 147.92 (.000) |
| + Social Risk | 14360 | -42583 | 11 | 85188 | 85272 | 235.85 (.000) |
| + smoke preg (full) | 14360 | -42415 | 13 | 84856 | 84956 | 335.68 (.000) |
| M4 v M2 | 14360 |  |  |  |  | 1995.95 (.000) |

**Table S.2b**

*Descriptive statistics for SDQ with and without winsorising (i.e. replacing scores of 0 with scores of 1 for gamma distribution)*

|  | Obs= | Mean (SD) | Med (IQR) | Range |
| --- | --- | --- | --- | --- |
| SDQ-total as observed | 14,360 | 7.61(5.35) | 7(6) | 0-39 |
| SDQ-total (532 zeros given a 1) | 14,360 | 7.64 (5.3) | 7(6) | 1-39 |

**Table S.3a**

*Demonstrating using goodness of fit statistics that GLMM with binomial distribution and log function was appropriate for the data*

|  | n= | loglikelihood | df | AIC | BIC | LRchi2(p-val) |
| --- | --- | --- | --- | --- | --- | --- |
| Null Model | 14360 | -3276 | 1 | 6554 | 6561 |  |
| +preg, no random (m2) | 14360 | -3240 | 5 | 6489 | 6527 | 72.87 (.000) |
| no preg, +random | 14360 | -3081 | 2 | 6165 | 6180 |  |
| + preg + random (m4) | 14360 | -3052 | 6 | 6116 | 6162 | 110.66 (.000) |
| + sex | 14360 | -3030 | 7 | 6074 | 6127 | 44.85 (.000) |
| + Social Risk | 14360 | -2958 | 10 | 5935 | 6012 | 144.24 (.000) |
| + smoke preg | 14360 | -2907 | 12 | 5837 | 5929 | 102.20 (.000) |
| M4 v M2 | 14360 |  |  |  |  | 374.60 (.000) |

**Table S.4**

*Predicted probabilities of being in clinical SDQ range based on different characteristics of predictors (for all smoking held constant at no people in household smoked in pregnancy.)*

|  | Sex | Total no. of Pregnancy complications | Social Risk | Predicted probability of being in the clinical range on SDQ (95%CI) |
| --- | --- | --- | --- | --- |
| 1. | Males | 4+ | High | 22.6% (15.4-29.7%) |
| 2. | Females | 4+ | High | 15.6%(10.0-21.2%) |
| 3. | Males | 0 | Low | 3.7%(2.9-4.5%) |
| 4. | Females | 0 | Low | 2.1%(1.6-2.6%) |
| 5. | Males | 0 | High | 9.0%(7.1-10.9%) |
| 6. | Females | 0 | High | 5.5%(4.2-6.9%) |
| 7. | Males | 1 | Low | 5.5%(4.4-6.7%) |
| 8. | Females | 1 | Low | 3.2%(2.5-4.0%) |

Note: “ideal types are particularly illustrative for interpretation when independent variables are substantially correlated” (Long & Freese, 2014).

**Table S.5** *Comparison of people included with excluded on exposure, outcome and covariates of interest.*

| Variable | Total (n=11,134) | Included (n=8,483) | Excluded (n=2,651) |
| --- | --- | --- | --- |
| Total number of pregnancy complications (%): | | | |
| 0 | 45.99 | 44.91 | 56.14 |
| 1 | 32.77 | 33.61 | 24.86 |
| 2 | 13.93 | 14.02 | 13.10 |
| 3 | 4.67 | 4.76 | 3.79 |
| 4+ | 2.65 | 2.70 | 2.12 |
| Maternal Age (M, SD): | 31.44 (5.43) | 31.41 (5.41) | 31.69 (5.55) |
| Mother in relationship  (% yes): | 83.84 | 83.90 | 83.25 |
| Mother NOT born in Ireland (% yes): | 19.47 | 18.95 | 24.50 |
| Maternal Education Level (%): | | | |
| MSc/PhD | 10.77 | 10.97 | 8.85 |
| Degree | 16.34 | 16.44 | 15.35 |
| Post-secondary up to diploma | 28.24 | 27.87 | 31.87 |
| None up to secondary school | 44.65 | 44.73 | 43.93 |
| ^a^ Family equivalised income quintile (%): | | | |
| High | 38.53 | 38.60 | 34.91 |
| Medium | 20.21 | 20.08 | 26.83 |
| Low | 41.26 | 41.31 | 38.26 |
| Households self-reported ability to make ends meet (%): | | | |
| With great difficulty | 4.82 | 4.80 | 4.97 |
| With difficulty | 8.12 | 8.22 | 7.16 |
| With some difficulty | 32.14 | 32.15 | 32.08 |
| Fairly easily | 37.29 | 37.17 | 38.42 |
| Easily | 13.28 | 13.35 | 12.62 |
| Very easily | 4.34 | 4.30 | 4.75 |
| ^b^ Cumulative social risk category (%): | | | |
| No risk | 14.49 | 14.55 | 10.93 |
| Low risk | 29.83 | 29.76 | 34.13 |
| Moderate risk | 31.40 | 31.44 | 29.21 |
| High risk | 24.28 | 24.25 | 25.73 |
| SDQ-total @5-years: | | | |
| Mean (SD) | 7.59 (5.00) | 7.58 (5.01) | 7.66 (4.94) |
| SDQ-total @9-years: | | | |
| Mean (SD) | 7.66 (5.68) | 7.64 (5.70) | 7.82 (5.45) |
| Number of people who smoked in the household during pregnancy: | | | |
| Mean (SD) | .46 (.72) | .47 (.72) | .42 (.68) |
| Child sex (% Male): | 51.32 | 51.05 | 53.78 |
| SDQ-categories @ 5-years (%): | | | |
| Average | 87.66 | 87.60 | 88.27 |
| Slightly raised | 6.40 | 6.50 | 5.29 |
| High | 3.09 | 3.10 | 2.95 |
| Very high | 2.86 | 2.80 | 3.50 |
| SDQ-categories @ 9-years (%): | | | |
| Average | 85.32 | 85.48 | 83.85 |
| Slightly raised | 6.35 | 6.21 | 7.63 |
| High | 4.06 | 3.99 | 4.82 |
| Very high | 4.26 | 4.32 | 3.70 |

^a^ Family Equivalised Income reflects that from all sources, after tax and social insurance divided by the number of people in the household. ^b^ Comprises maternal age, education, relationship, migrancy and family income.

**Table S.6**

*Goodness of fit tests for adding interaction term to model*

|  | n= | loglikelihood | df | AIC | BIC | LRchi2(p-val) |
| --- | --- | --- | --- | --- | --- | --- |
| Full Model | 14360 | -42415 | 13 | 84856 | 84956 |  |
| +preg##sex | 14360 | -42412 | 17 | 84858 | 84988 | 6.45 (.168) |
| Full Model | 14360 | -42415 | 13 | 84856 | 84956 |  |
| + preg##Social risk | 14360 | -42410 | 25 | 84870 | 85061 | 9.75 (.638) |

**Table S.7**

*Association between pregnancy complications, sex and social risk with odds of being in the clinical SDQ category.*

|  | Odds Ratio (95% CI) |
| --- | --- |
| No complications | REF |
| 1 complication | 1.88 (1.37-2.59) |
| 2 complications | 2.31 (1.53-3.51) |
| 3 complications | 1.77 (.89- 3.52) |
| 4+ complications | 6.88 (3.29-14.40) |
| Male | REF |
| Female | .43 (.32-.57) |
| No social risk | REF |
| Low social risk | 1.56 (1.00-2.43) |
| Moderate social risk | 2.84 (1.81- 4.46) |
| High social risk | 6.83 (4.16-11.22) |
| No smoke in pregnancy | REF |
| Exposure to smoke in pregnancy | 1.49 (1.24-1.78) |

**Table S.8**

*Fully-adjusted model with the addition of controlling for postnatal depression (as measured by CES-D score of mother at 9-months) as sensitivity analysis*

|  | Effect estimates and 95% CI’s after adjustment | | | | | | |
| --- | --- | --- | --- | --- | --- | --- | --- |
|  | Total | Stratified by sex | | Stratified by social risk | | | |
| Total no.of ^a^PCs |  | Male  (n=3677) | Female  (n=3658) | No risk | Low risk | Mod risk | High risk |
| None | Reference | | | | | | |
| 1 | 1.08(1.05-1.12) | 1.11(1.06-1.16) | 1.06(1.01-1.12) | 1.11(1.03-1.20) | 1.04(.99-1.11) | 1.08(1.01-1.15) | 1.15(1.05-1.26) |
| 2 | 1.17(1.12-1.22) | 1.22(1.14-1.31) | 1.12(1.05-1.19) | 1.27(1.14-1.41) | 1.18(1.09-1.27) | 1.12(1.03-1.22) | 1.13(1.01-1.26) |
| 3 | 1.17(1.10-1.25) | 1.20(1.09-1.33) | 1.13(1.02-1.24) | 1.26(1.06-1.51) | 1.13(1.01-1.28) | 1.14(1.01-1.29) | 1.17(1.00-1.37) |
| 4+ | 1.27(1.15-1.40) | 1.37(1.18-1.59) | 1.19(1.04-1.36) | 1.17(.90-1.53) | 1.23(1.03-1.47) | 1.20(.98-1.47) | 1.42(1.19-1.70) |

^a^ Pregnancy complications
